# Association between Empirical Anti-Pseudomonal Antibiotics and Progression to Thoracic Surgery and Death in Empyema: Database Research

**DOI:** 10.3390/antibiotics13050383

**Published:** 2024-04-24

**Authors:** Akihiro Shiroshita, Kentaro Tochitani, Yohei Maki, Takero Terayama, Yuki Kataoka

**Affiliations:** 1 Division of Epidemiology, Department of Medicine, Vanderbilt University School of Medicine, Nashville, TN 37203, USA; 2 Scientific Research Works Peer Support Group (SRWS-PSG), Osaka 541-0043, Japan; 3 Department of Environmental Health, Harvard T.H. Chan School of Public Health, Boston, MA 02115, USA; 4 Department of Infectious Diseases, Kyoto City Hospital, Kyoto 604-8845, Japan; 5 Division of Infectious Diseases and Respiratory Medicine, National Defense Medical College, Saitama 359-8513, Japan; 6 Division of Infectious Diseases, Massachusetts General Hospital, Boston, MA 02114, USA; 7 Department of Emergency, Self-Defense Forces Central Hospital, Tokyo 154-8532, Japan; 8 Department of Internal Medicine, Kyoto Min-Iren Asukai Hospital, Kyoto 616-8147, Japan; 9 Section of Clinical Epidemiology, Department of Community Medicine, Kyoto University Graduate School of Medicine, Kyoto 606-8507, Japan; 10 Department of Healthcare Epidemiology, Kyoto University Graduate School of Medicine/Public Health, Kyoto 606-8501, Japan

**Keywords:** empyema, antibiotics, thoracic surgery, mortality, database research

## Abstract

Evidence on the optimal antibiotic strategy for empyema is lacking. Our database study aimed to evaluate the effectiveness of empirical anti-pseudomonal antibiotics in patients with empyema. We utilised a Japanese real-world data database, focusing on patients aged ≥40 diagnosed with empyema, who underwent thoracostomy and received intravenous antibiotics either upon admission or the following day. Patients administered intravenous vasopressors were excluded. We compared thoracic surgery and death within 90 days after admission between patients treated with empirical anti-pseudomonal and non-anti-pseudomonal antibiotics. Cause-specific hazard ratios for thoracic surgery and death were estimated using Cox proportional hazards models, with adjustment for clinically important confounders. Subgroup analyses entailed the same procedures for patients exhibiting at least one risk factor for multidrug-resistant organisms. Between March 2014 and March 2023, 855 patients with empyema meeting the inclusion criteria were enrolled. Among them, 271 (31.7%) patients received anti-pseudomonal antibiotics. The Cox proportional hazards models indicated that compared to empirical non-anti-pseudomonal antibiotics, empirical anti-pseudomonal antibiotics were associated with higher HRs for thoracic surgery and death within 90 days, respectively. Thus, regardless of the risks of multidrug-resistant organisms, empirical anti-pseudomonal antibiotics did not extend the time to thoracic surgery or death within 90 days.

## 1. Introduction

Empyema is a pleural infection characterised by the accumulation of pus within the thoracic cavity. Drainage is the cornerstone of treatment, with thoracostomy or percutaneous chest tube drainage being the first choice [1,2,3]. Simultaneously, physicians promptly initiate empirical antibiotics following the empyema diagnosis. In cases where initial treatment fails, patients may undergo intrapleural fibrinolytic and/or thoracic surgery, such as video-assisted thoracic surgery and open thoracotomy. While previous and current randomised controlled studies have focused on thoracic surgery and intrapleural fibrinolytic therapy, optimal antibiotic strategies lack sufficient data [4,5].

International guidelines recommend selecting antibiotics based on the empyema onset site (i.e., community- vs. hospital-acquired), underlying medical conditions, and local bacterial characteristics. However, supporting evidence predominantly stems from expert opinions, bacterial susceptibility tests, and pneumonia studies [1,2,3]. Moreover, the empirical antibiotics selection remains contentious across diseases, with some studies indicating that broad-spectrum antibiotics might elevate mortality rates, even among patients prone to multidrug-resistant organisms [6,7].

To the best of our knowledge, no study has evaluated the effectiveness of empirical anti-pseudomonal antibiotics for empyema. Thus, in this study, we aimed to elucidate antibiotic strategies in real-world practice across hospitals to evaluate whether empirical anti-pseudomonal antibiotics improve patient outcomes in empyema. In addition, we evaluated the effectiveness of empirical anti-pseudomonal antibiotics among patients with risk factors for multidrug-resistant organisms.

## 2. Results

### 2.1. Descriptive Analysis

**Figure 1:**
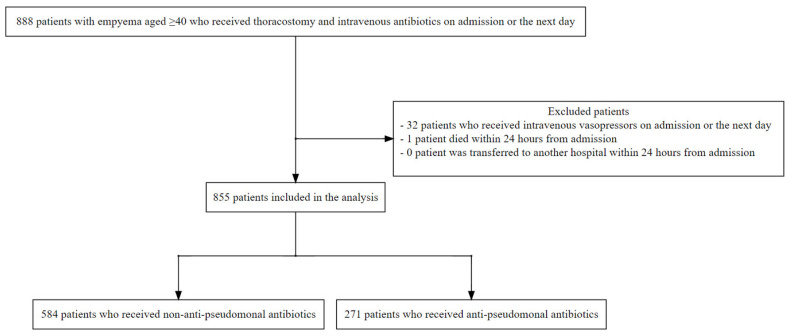
Patient selection flow. We selected 888 patients with empyema aged ≥ 40 years who underwent thoracostomy and received intravenous antibiotics upon admission or the next day. Among them, 32 patients received intravenous vasopressors upon admission or the next day, and 1 died within 24 h from admission. After excluding these patients, 855 patients were included in the analysis.

Between March 2014 and March 2023, we identified 888 patients with empyema aged ≥ 40 years who underwent thoracostomy and were administered intravenous antibiotics upon admission or the following day (Figure 1). Among them, 32 patients were administered intravenous vasopressors upon admission or the next day, and 1 died within 24 h of admission. After excluding these patients, ultimately 855 patients were included in the analysis. Patient characteristics are summarised in Table 1. The distribution of each baseline variable was similar between the two groups. Regarding empirical antibiotics, 271 (31.7%) patients received anti-pseudomonal antibiotics, while 584 (68.3%) received non-anti-pseudomonal antibiotics. The most frequently prescribed agents were piperacillin/tazobactam (189/271 [69.7%]) and ampicillin/sulbactam (546/584 [93.5%]) (Table 2). Methicillin-resistant Staphylococcus aureus (MRSA) was empirically covered in nine cases. Overall, 62/855 (7.3%) patients had missing covariates. A total of 382 patients (empirical anti-pseudomonal antibiotic group: 141 vs. empirical non-anti-pseudomonal antibiotic group: 241) had at least one risk factor for multidrug-resistant organisms. 

Among the included patients, 19/888 (2.1%) patients were assigned the disease name of ‘empyema due to MRSA.’ No other disease names related to specific bacteria were identified. Some hospitals, especially small- and medium-sized hospitals, did not store bacterial culture results in their databases (Table S3). Although the bacterial culture of pleural fluid was ordered for 690/855 (80.7%) patients, pleural culture results were obtained only for 171/855 (20.0%) patients. Among them, we found no case of *Pseudomonas aeruginosa* but four cases of MRSA and one drug-resistant Gram-negative bacterium that were not susceptible to at least one of the anti-pseudomonal antibiotics. In contrast, de-escalation from anti-pseudomonal antibiotics to non-anti-pseudomonal antibiotics within 7 days was performed in 12/33 (36.3%) patients. 

**Figure 2:**
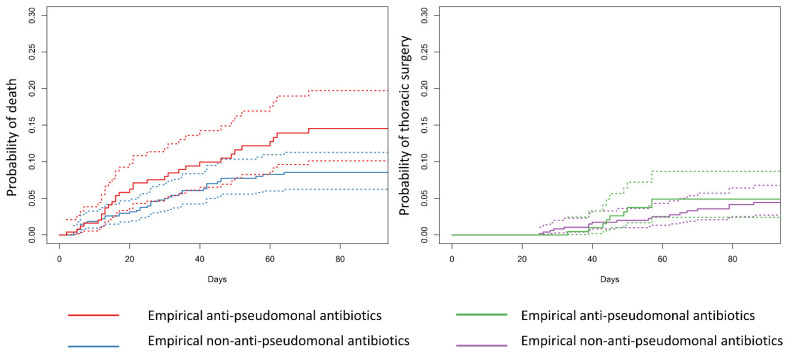
Cumulative incidence functions of thoracic surgery and death. The curves illustrate the cumulative incidence functions of thoracic surgery and death among the empirical anti-pseudomonal and empirical non-anti-pseudomonal antibiotics groups, respectively. These curves represent estimates of actual probabilities that a patient has for each event. We did not adjust confounders in this figure. The solid lines represent the point estimates, and the dotted lines represent the 95% confidence interval.

The median length of stay was 28.0 days (IQR: 18.0–40.5 days) and 23.0 days (IQR: 17.0, 34.0 days) among empirical anti-pseudomonal and empirical non-anti-pseudomonal antibiotics groups, respectively. In the empirical anti-pseudomonal group, 35 (12.9%) patients underwent thoracic surgery and 32 (11.8%) died; in the empirical non-anti-pseudomonal group, 54 (9.2%) patients underwent thoracic surgery and 47 (8.0%) died 90 days after admission. The cumulative incidence functions showed that before adjusting for confounders, the risks of thoracic surgery and death tended to be higher in the empirical anti-pseudomonal antibiotic group than in the empirical non-anti-pseudomonal antibiotic group (Figure 2). 

**Figure 3:**
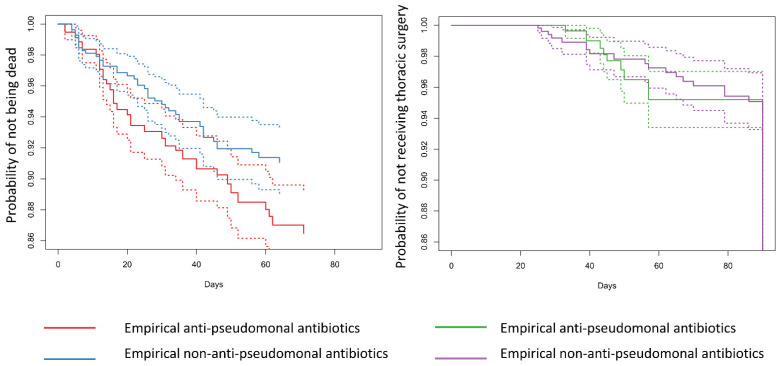
The estimated survival function of the main and subgroup analyses. The main analysis was the Cox proportional hazards model estimating the cause-specific hazard ratio of empirical anti-pseudomonal antibiotics compared to empirical non-anti-pseudomonal antibiotics on thoracic surgery and death within 90 days. In the subgroup analysis, we selected patients who had at least one of the following risk factors for the presence of multidrug-resistant organisms: residence in a healthcare facility, dialysis, previous antibiotic use within 90 days, or immunodeficiency. Clinically meaningful confounders were adjusted by using inverse probability weighting. We estimated the survival function based on the four Cox proportional hazards models. The solid lines represent the point estimates, and the dotted lines represent the 95% confidence interval. Both analyses showed anti-pseudomonal antibiotics increased the risk of death within 90 days.

The results of the statistical analysis are summarised in Table 3. After propensity score weighting, the distributions of the covariates were similar between the groups (Figure S1). Table 3 summarises the results of the weighted Cox proportional hazards models, and Figure 3 shows the estimated survival functions. Empirical anti-pseudomonal antibiotics were associated with a higher HR of death within 90 days compared to empirical non-anti-pseudomonal antibiotics both in the main and subgroup analyses (main analysis: HR: 1.52 [95% CI: 0.94–2.44]; subgroup analysis: 2.06 [95% CI: 1.03–4.13]). Although the Cox proportional hazard models indicated that empirical anti-pseudomonal antibiotics were associated with a higher risk of thoracic surgery (main analysis: HR: 1.63 [95% CI: 1.05–2.54]; and subgroup analysis: HR: 1.45 [95% CI: 0.72–2.93]), the estimated survival curves intersected. Exploratory analyses did not demonstrate any clear connections (Table 3). 

### 2.2. Bias Analysis

Figure S2 summarises the results of biased analyses. We simulated different pairs of bias parameters regarding the effect estimates of empirical anti-pseudomonal antibiotics on the 90-day mortality among high-risk groups for multidrug-resistant organisms. The results consistently showed harmful point estimates, and although the confidence intervals crossed the nonsignificant threshold of 1, none of the simulation results showed a protective association with 90-day mortality.

## 3. Discussion

To date, evidence on the initial antibiotic selection for empyema is lacking. Our study revealed that anti-pseudomonal antibiotics were empirically administered in 31.7% of the patients with empyema who did not receive intravenous vasopressors upon admission. Furthermore, after adjusting for numerous known confounders, we found no extension in time to death and thoracic surgery within 90 days, irrespective of the risk of multidrug-resistant bacteria. Additionally, our bias analyses did not support the effectiveness of empirical anti-pseudomonal antibiotics on the 90-day mortality rates.

Our results suggest that when a patient with empyema is not in shock and undergoes thoracostomy upon admission, physicians may not need to administer anti-pseudomonal antibiotics empirically, regardless of the risk of multidrug-resistant organisms. While our main and subgroup analyses indicated a potentially harmful effect of empirical anti-pseudomonal antibiotics, unmeasured confounders related to the severity and risk of multidrug-resistant organisms may have skewed the effect estimates in a harmful direction. Therefore, we simulated various situations in which additional strong confounding factors existed. Even in the presence of an additional confounder, empirical anti-pseudomonal antibiotics did not demonstrate a protective effect of among those who were at risk of multidrug-resistant organisms. In general, narrow-spectrum antibiotics are associated with fewer subsequent infections, fewer adverse reactions, and lower costs than broad-spectrum antibiotics; therefore, empirical non-anti-pseudomonal antibiotics should be a reasonable first choice [6,8,9].

Furthermore, our study highlights the low frequency of antibiotic de-escalation in patients with empyema. Although we obtained bacterial culture results in only 20.0% of the patients, it was notable that among the empirical anti-pseudomonal antibiotics group, 36.3% of the patients continuously received anti-pseudomonal antibiotics without detecting drug-resistant organisms during hospitalisation. This may be because drainage is the mainstay treatment for empyema, and physicians may not pay attention to antibiotic strategies. Additionally, an extremely low number of drug-resistant organisms were detected in our study, consistent with our previous multicentre retrospective cohort study in tertiary-care settings in Japan, where *Pseudomonas aeruginosa* and extended-spectrum β-lactamase-producing Enterobacterales were detected in <1% of the included patients [10]. Thus, our study underscores the need for local epidemiological research specific to empyema. Given the low sensitivity of pleural effusion bacterial culture, future studies could benefit from utilizing genetic tests such as the amplification and sequencing of the bacterial 16S ribosomal RNA gene [11]. Moreover, the prevalence of multidrug-resistant organisms is a global concern; therefore, physicians should avoid the unnecessary use of broad-spectrum antibiotics [12]. Nevertheless, further studies are required to identify subgroups with high risk for drug-resistant organisms, specifically empyema, to avoid the emergence of multidrug-resistant organisms.

Although this study has clinical and research implications, it had some limitations. First, the study population included Japanese patients with empyema. However, previous research in Europe and the United States estimated a 5–10% prevalence of *Pseudomonas aeruginosa* in empyema [12,13,14]. Additionally, empirical antibiotics should be selected based on their severity (e.g., blood pressure and oxygen demand), risks for drug-resistant organisms, and local epidemiology [15]. Therefore, external validation studies in different countries are required to confirm the validity of our results. Second, our study did not consider the stage of empyema or imaging findings such as loculation, septation, lung abscess, and fistula, which may be predictors of bad outcomes [16,17,18]. However, we expected that these factors would not influence the choice of antibiotics, and the confounding factors would be small. Finally, we were unable to evaluate other important outcomes related to patient quality of life, such as an unexpandable lung.

In conclusion, our study showed that in real-world data, regardless of the risk of multidrug-resistant organisms, empirical anti-pseudomonal antibiotics did not extend the time to thoracic surgery and death within 90 days. Further RCTs are required to address residual confounding factors and evaluate the causal relationship between the empirical use of anti-pseudomonal antibiotics and each outcome.

## 4. Materials and Methods

### 4.1. Study Design

We used a large-scale dataset from Japan, the RWD database. This database is maintained by the Health, Clinic, and Education Information Evaluation Institute (HCEI, Kyoto, Japan) and with support from JMDC Inc. (Tokyo, Japan). This included electronic medical records (e.g., demographic, pharmacy, and laboratory data) and administrative claims data (e.g., disease name and procedure data) of approximately 20 million patients from over 200 medical institutions in Japan. This study was conducted in accordance with the Declaration of Helsinki. The institutional review Board of Showa University approved this study (approval number: 2023-119-B). The requirement for written informed consent was waived, owing to the retrospective nature of the study. This article has been reported in accordance with the REporting of studies Conducted using Observational Routinely collected Data statement (Table S1) [19].

### 4.2. Patient Selection

Our target population was patients aged ≥ 40 years who were diagnosed with empyema before admission (community-acquired and hospital-acquired empyema) and underwent a thoracostomy and were administered intravenous antibiotics upon admission or the following day. The International Statistical Classification of Diseases and Related Health Problems (ICD-10) J86 was used to identify patients with empyema. Our previous study validated the accuracy of the ICD-10 codes in detecting patients with empyema (positive predictive value, 83%) [11]. Patients who received intravenous vasopressors upon admission or the following day were excluded due to their likely need for broad-spectrum antibiotics. The definitions of intravenous vasopressors are summarised in Table S2. Additionally, those who died or were transferred to another hospital within 24 h were excluded.

### 4.3. Data Extraction

We extracted patient demographic characteristics and diagnoses from the EF1 files submitted to the government for the reimbursement of medical fees. These included age, admission date, department, route of admission (home, nursing home, or hospital), sex, body mass index, smoking status (Brinkman index), exercise tolerability (Hugh-Johns classification), the activities of daily living (ADLs; Barthel index), comorbidities, oxygen use upon admission, mental status upon admission, discharge date, and prognosis [20]. Comorbidities were supplemented by reviewing outpatient claims within 90 days of admission to the same hospital. In addition, we extracted procedure and drug prescription data from claims and laboratory and bacterial culture data from the data warehouse at each hospital. The coding dictionary is summarised in Table S2.

### 4.4. Exposure

We selected intravenous anti-pseudomonal antibiotics administered upon admission or the next day as the intervention, regardless of the dose. These definitions are summarised in Table S2. The comparator was an intravenous non-anti-pseudomonal antibiotic.

### 4.5. Outcome

The primary outcomes were thoracic surgery (K-code: K488-3, K488-4, K496-2, K496-4, and K515) and death, regardless of cause, within 90 days after admission. The RWD database enabled us to longitudinally follow up patients at the same hospital using electronic medical records. If a patient was lost to follow-up within 90 days, it was censored. For exploratory analyses, the following outcomes were compared between the two groups: intravenous vasopressors tracheal intubation and mechanical ventilation within 7 days from admission, the outcome at discharge, and the proportion of *Clostridioides difficile* colitis that was defined as ICD-10 code A047 during hospitalisation (electrical medical records or Yoshiki1) or use of oral vancomycin during hospitalisation.

### 4.6. Covariates

For the statistical analysis, we used propensity score weighting (inverse probability of treatment weight) to receive empirical anti-pseudomonal antibiotics. The propensity score was calculated using multivariable logistic regression with the following clinically meaningful covariates: age (continuous), sex, ADLs (full support or partially dependent [Barthel index < 50] and non-full-dependent [Barthel index ≥ 50]), exercise tolerability (high: Hugh-Johns classification ≤ 3, low: Hugh-Johns classification > 3), immunodeficiency (malignancy or use of systemic steroid or immunosuppressive agents within 90 days before admission), oral or intravenous antibiotics use within 90 days before admission, source of infection (community-acquired or hospital-/nursing care-acquired), mental status (normal [Japan Comma Scale = 0] and abnormal [Japan Comma Scale > 0]), blood urea nitrogen (low: <14 mg/dL, moderate: ≥14–<22.4 mg/dL, high: ≤22.4 mg/dL), serum albumin (low: ≤2.7 g/dL, high: >2.7 g/dL), and oxygen use (presence or absence) [12,13,21,22,23,24]. Using propensity score weighting, we resembled the distribution of confounders in each group to the overall study population (average treatment effect). After assigning propensity score weights to each patient, we visually confirmed the balance of the covariates between the anti-pseudomonal and non-anti-pseudomonal antibiotic groups.

### 4.7. Statistical Analysis

We summarised patient characteristics by exposure (empirical anti-pseudomonal antibiotic group vs. empirical non-anti-pseudomonal antibiotic group) as frequencies and proportions for categorical variables and means with standard deviations or medians with interquartile ranges (IQRs) for continuous variables. Additionally, we plotted the cumulative incidence functions for thoracic surgery and death.

As death precludes the observation of thoracic surgery (competing risk), we estimated the cause-specific hazard ratio, which quantifies the risk of an event in a population where the competing risk is removed [25]. The cause-specific hazard ratio (HR) is a valid estimate of the relative change in the causal inference framework. Utilizing the Cox proportional hazards model, we estimated the cause-specific HRs and depicted the estimated survival functions for each treatment group. A robust standard error was used to calculate 95% confidence intervals (CIs). As a subgroup analysis, we evaluated the effectiveness of empirical anti-pseudomonal antibiotics in patients who had at least one of the following potential risk factors for the presence of multidrug-resistant organisms: residence in a healthcare facility, dialysis, previous antibiotic use within 90 days, or immunodeficiency [15,26,27]. Furthermore, all confounders other than the risks for multidrug-resistant organisms were incorporated into a propensity score, and the same methodology as used in the main analysis was applied. We compared the exploratory outcomes using the Chi-squared test.

We performed probabilistic bias analyses to simulate the extent to which our estimate in the subgroup analysis would be skewed, owing to an unmeasured strong binary confounder because there could be an unmeasured confounder [28,29]. The following steps were performed:

1. A specific pair of bias parameters was set: the prevalence of a binary unmeasured confounder *C* among the empirical anti-pseudomonal antibiotics (E=1) group ($p_{1}=\{0.4,\;0.5,\;0.6\}$) and the empirical non-anti-pseudomonal (E=0) antibiotics group ($p_{0}=\{0.1,\;0.2,\;0.3\}$) and risk ratio of *C* and death at 90 days (D), that is $RR_{cd}=\{3.0,\;4.0,\;5.0\}$. These $RR_{cd}$ values were set based on the risk ratios between the measured confounders and death (median: 3.2; IQR: 2.0–3.5).
2. Probability distribution was assigned to each bias parameter to consider the uncertainty of the bias parameter: $p_{1}∼Beta(\alpha_{1},\;\beta_{1})$, $p_{0}∼Beta(\alpha_{0},\;\beta_{0})$, and $p_{0}∼Trapezoidal(RR_{cd}-0.2,\;RR_{cd}-0.1,RR_{cd}+0.1,RR_{cd}+0.2)$ where α and β parameters were defined based on the mean ($p_{1}$ and $p_{0}$) and their plausible 2.5–9.5th percentile ($p_{1}\pm 0.1$ and $p_{0}\pm 0.1$).
3. A random sample of bias parameters from the specified distributions in Step 2 was used.
4. The probability of having a confounder within the levels of treatment and outcome was calculated: $\mathrm{Pr}\left(C+|E+,\;D+\right)$, $\mathrm{Pr}\left(C+|E-,\;D+\right)$, $\mathrm{Pr}\left(C+|E+,\;D-\right)$, and $\mathrm{Pr}\left(C+|E-,\;D-\right)$.
5. Bernoulli distribution was assigned to $\mathrm{Pr}\left(C+|E+,\;D+\right)$, $\mathrm{Pr}\left(C+|E-,\;D+\right)$, $\mathrm{Pr}\left(C+|E+,\;D-\right)$, and $\mathrm{Pr}\left(C+|E-,\;D-\right)$ to consider the uncertainty.
6. The probability of having a confounder from the specified distribution in step 4 was randomly sampled.
7. A new column of C in the original dataset was created.
8. The same subgroup analysis with robust standard error was performed.
9. Calculating a bias-adjusted estimate was calculated.
10. Steps 1–9 were repeated for 100.000 iterations and the median and 95% CI were categorize.

The results of the bias analyses were categorized as forest plots using R version 4.3.2 (R Foundation, Vienna, Austria) for all statistical analyses. Statistical significance was set at *p* < 0.05 significant. We performed a complete case analysis because the amount of missing data was quite small. 

## Figures and Tables

**Table 1:** Patient characteristics.

	Empirical Non-Anti-Pseudomonal Antibiotics (N ^a^ = 584)	Empirical Anti-Pseudomonal Antibiotics (N = 271)	Overall (N = 855)
Age (mean (SD ^‡^))	75.5 (12.5)	74.7 (11.8)	75.2 (12.3)
Male	463 (79.3)	217 (80.1)	680 (79.5)
Number of beds (%)			
≥100–<300	59 (10.1)	28 (10.3)	87 (10.2)
≥300–<500	239 (40.9)	121 (44.6)	360 (42.1)
≥500	286 (49.0)	122 (45.0)	408 (47.7)
Source of infection (%)			
Community-acquired	468 (80.1)	208 (76.8)	676 (79.1)
Nursing care-acquired	51 (8.7)	20 (7.4)	71 (8.3)
Hospital-acquired	65 (11.1)	43 (15.9)	108 (12.6)
Body mass index (%)			
<18.5 kg/m^2^	114 (19.5)	74 (27.3)	188 (22.0)
≥18.5–<25 kg/m^2^	275 (47.1)	118 (43.5)	393 (46.0)
≥25 kg/m^2^	89 (15.2)	44 (16.2)	133 (15.6)
Missing	106 (18.2)	35 (12.9)	141 (16.5)
Activity of daily living (%)			
Full support	132 (22.6)	79 (29.2)	211 (24.7)
Partially dependent	84 (14.4)	28 (10.3)	112 (13.1)
Independent	368 (63.0)	164 (60.5)	532 (62.2)
Altered mental status (%)	131 (22.4)	67 (24.7)	198 (23.2)
Missing	4 (0.7)	4 (1.5)	8 (0.9)
Exercise tolerability (%)			
Low	214 (36.6)	89 (32.8)	303 (35.4)
Missing	3 (0.5)	3 (1.1)	6 (0.7)
Immunodeficiency (%)	127 (21.7)	84 (31.0)	211 (24.7)
Home oxygen therapy (%)	8 (1.4)	4 (1.5)	12 (1.4)
Smoking (%)	340 (58.2)	161 (59.4)	501 (58.6)
Charlson Comorbidity Score (median [IQR ^§^])	4.0 [3.0, 6.0]	4.0 [3.0, 6.0]	4.0 [3.0, 6.0]
Previous antibiotics use within 90 days before admission	94 (16.1)	54 (19.9)	148 (17.3)
Dialysis at baseline (%)	3 (0.5)	2 (0.7)	5 (0.6)
Blood urea nitrogen (%)			
<14 mg/dL	208 (35.6)	99 (36.5)	307 (35.9)
≥14–<22.4 mg/dL	209 (35.8)	88 (32.5)	297 (34.7)
≤22.4 mg/dL	162 (27.7)	81 (29.9)	243 (28.4)
Missing	5 (0.9)	3 (1.1)	8 (0.9)
Serum albumin (%)			
≤2.7 g/dL	90 (15.4)	28 (10.3)	118 (13.8)
Missing	31 (5.3)	16 (5.9)	47 (5.5)
Oxygen use on admission (%)	361 (61.8)	169 (62.4)	530 (62.0)

Abbreviations: ^a^: N = number, ^‡^: SD = standard deviation, ^§^: IQR = interquartile range.

**Table 2:** List of empirical antibiotics used in the included patients.

Antibiotics	Frequency (%)
Anti-pseudomonal antibiotics (N * = 287)	
Piperacillin/tazobactam	189 (65.9)
Carbapenem	79 (27.5)
Quinolone	7 (2.4)
Other antibiotics	12 (4.2)
Non-anti-pseudomonal antibiotics (N = 568)	
Ampicillin/sulbactam	546 (96.1)
Ceftriaxone	60 (10.6)
Clindamycin	33 (5.8)
Vancomycin	9 (1.6)
Metronidazole	3 (0.5)

Abbreviations: *: N = number.

**Table 3:** Summary of statistical analyses.

	Empirical Anti-Pseudomonal Antibiotics (N * = 271)	Empirical Non-Anti-Pseudomonal Antibiotics (N = 584)	*p*-Value
Death within 90 days from admission (%)			
Alive	132 (48.7)	312 (55.0)	
Death	32 (11.8)	47 (8.0)	
Censored	107 (39.5)	216 (37.0)	
Thoracic surgery within 90 days from admission (%)			
No thoracic surgery	106 (39.1)	285 (48.8)	
Thoracic surgery	35 (12.9)	54 (9.2)	
Censored	130 (48.0)	245 (42.0)	
Main analyses (N = 793)			
Thoracic surgery	HR ^‡^: 1.63 (95% CI ^§^: 1.05–2.54)		0.891
Death	HR: 1.52 (95% CI: 0.94–2.44)		0.420
Subgroup analyses (N = 352)			
Thoracic surgery	HR: 1.45 (95% CI: 0.72–2.93)		0.508
Death	HR: 2.06 (95% CI: 1.03–4.13)		0.040
Exploratory analyses			
Intravenous vasopressor within 7 days from admission	8 (3.0)	6 (1.0)	0.076
Intrapleural urokinase therapy during hospitalization	97 (35.8)	164 (28.1)	0.028
Tracheal intubation within 7 days from admission	1 (0.2)	3 (1.1)	0.219
Mechanical ventilation within 7 days from admission	14 (2.4)	10 (3.7)	0.400
Outcome at discharge (%)			0.780
Discharge	262 (96.7)	565 (96.7)	
Transferred to another hospital	9 (3.3)	18 (3.1)	
In-hospital death	0 (0.0)	0 (0.0)	
Missing	0 (0.0)	1 (0.2)	
*Clostridioides difficile* colitis (%)	6 (2.2)	17 (2.9)	0.720

Abbreviations: *: N = number, ^‡^: HR = hazard ratio, ^§^: CI = confidence interval.

## Data Availability

The data supporting the findings of this study are available upon request from the corresponding author. The data are not publicly available because of restrictions imposed by Real World Data Co., Ltd.

## Supplementary Materials

The following supporting information can be downloaded at: https://www.mdpi.com/article/10.3390/antibiotics13050383/s1, Table S1: The RECORD statement; Table S2: Variable definitions; Table S3: Patient characteristics stratified by the presence of bacterial culture results; Figure S1: Balance check after the assignment of propensity score weight; and Figure S2: Bias analysis results.
