# Supplementary material for: Association between Empirical Anti-Pseudomonal Antibiotics and Progression to Thoracic Surgery and Death in Empyema: Database Research
Source: Antibiotics (Basel). 2024 Apr 24;13(5):383. doi: 10.3390/antibiotics13050383 (PMC11117277; doi:10.3390/antibiotics13050383)
Supplement: Supplementary file 1 [file antibiotics-13-00383-s001.zip › antibiotics-2950562-supplementary.pdf]

Supplementary Table S1. The RECORD statement

	Item No.	STROBE items	Location in manuscript where items are reported	RECORD items	Location in manuscript where items are reported
**Title and abstract**					
	1	(a) Indicate the study's design with a commonly used term in the title or the abstract (b) Provide in the abstract an informative and balanced summary of what was done and what was found	1	RECORD 1.1: The type of data used should be specified in the title or abstract. When possible, the name of the databases used should be included.<br><br>RECORD 1.2: If applicable, the geographic region and timeframe within which the study took place should be reported in the title or abstract.<br><br>RECORD 1.3: If linkage between databases was conducted for the study, this should be clearly stated in the title or abstract.	1
**Introduction**					
Background rationale	2	Explain the scientific background and rationale for the investigation being reported	2		
Objectives	3	State specific objectives, including any prespecified hypotheses	2		

Methods					
Study Design	4	Present key elements of study design early in the paper			
Setting	5	Describe the setting, locations, and relevant dates, including periods of recruitment, exposure, follow-up, and data collection	7-8		
Participants	6	*(a) Cohort study* - Give the eligibility criteria, and the sources and methods of selection of participants. Describe methods of follow-up<br><br>*Case-control study* - Give the eligibility criteria, and the sources and methods of case ascertainment and control selection. Give the rationale for the choice of cases and controls<br><br>*Cross-sectional study* - Give the eligibility criteria, and the sources and methods of selection of participants<br><br>*(b) Cohort study* - For matched studies, give matching criteria and number of exposed and unexposed	8	RECORD 6.1: The methods of study population selection (such as codes or algorithms used to identify subjects) should be listed in detail. If this is not possible, an explanation should be provided.<br><br>RECORD 6.2: Any validation studies of the codes or algorithms used to select the population should be referenced. If validation was conducted for this study and not published elsewhere, detailed methods and results should be provided.<br><br>RECORD 6.3: If the study involved linkage of databases, consider use of a flow diagram or other graphical display to demonstrate the data linkage process, including the number of individuals with linked data at each stage.	8

		Case-control study - For matched studies, give matching criteria and the number of controls per case			
Variables	7	Clearly define all outcomes, exposures, predictors, potential confounders, and effect modifiers. Give diagnostic criteria, if applicable.	8	RECORD 7.1: A complete list of codes and algorithms used to classify exposures, outcomes, confounders, and effect modifiers should be provided. If these cannot be reported, an explanation should be provided.	8
Data sources/ measurement	8	For each variable of interest, give sources of data and details of methods of assessment (measurement).<br><br>Describe comparability of assessment methods if there is more than one group	8		
Bias	9	Describe any efforts to address potential sources of bias	8-9		
Study size	10	Explain how the study size was arrived at	8		
Quantitative variables	11	Explain how quantitative variables were handled in the analyses. If applicable, describe which groupings were chosen, and why	8-9		
Statistical methods	12	(a) Describe all statistical methods, including those used to control for confounding	9		

		(b) Describe any methods used to examine subgroups and interactions<br><br>(c) Explain how missing data were addressed<br><br>(d) *Cohort study* - If applicable, explain how loss to follow-up was addressed<br><br>*Case-control study* - If applicable, explain how matching of cases and controls was addressed<br><br>*Cross-sectional study* - If applicable, describe analytical methods taking account of sampling strategy<br><br>(e) Describe any sensitivity analyses			
Data access and cleaning methods		..		RECORD 12.1: Authors should describe the extent to which the investigators had access to the database population used to create the study population.<br><br>RECORD 12.2: Authors should provide information on the data cleaning methods used in the study.	11
Linkage		..		RECORD 12.3: State whether the study included person-level, institutional-level, or other data linkage across two or more databases. The methods of linkage and	Not applicable

				methods of linkage quality evaluation should be provided.	
**Results**					
Participants	13	(a) Report the numbers of individuals at each stage of the study (*e.g.*, numbers potentially eligible, examined for eligibility, confirmed eligible, included in the study, completing follow-up, and analysed)<br>(b) Give reasons for non-participation at each stage.<br>(c) Consider use of a flow diagram	2	RECORD 13.1: Describe in detail the selection of the persons included in the study (*i.e.,* study population selection) including filtering based on data quality, data availability and linkage. The selection of included persons can be described in the text and/or by means of the study flow diagram.	2
Descriptive data	14	(a) Give characteristics of study participants (*e.g.*, demographic, clinical, social) and information on exposures and potential confounders<br>(b) Indicate the number of participants with missing data for each variable of interest<br>(c) *Cohort study* - summarise follow-up time (*e.g.*, average and total amount)	2-4		
Outcome data	15	*Cohort study* - Report numbers of outcome events or summary measures over time	5		

		Case-control study - Report numbers in each exposure category, or summary measures of exposure<br><br>*Cross-sectional study* - Report numbers of outcome events or summary measures			
Main results	16	(a) Give unadjusted estimates and, if applicable, confounder-adjusted estimates and their precision (e.g., 95% confidence interval). Make clear which confounders were adjusted for and why they were included<br><br>(b) Report category boundaries when continuous variables were categorized<br><br>(c) If relevant, consider translating estimates of relative risk into absolute risk for a meaningful time period	5		
Other analyses	17	Report other analyses done—e.g., analyses of subgroups and interactions, and sensitivity analyses	6		
**Discussion**					
Key results	18	Summarise key results with reference to study objectives	6		
Limitations	19	Discuss limitations of the study, taking into account sources of	7	RECORD 19.1: Discuss the implications of using data that were not created or	7

		potential bias or imprecision. Discuss both direction and magnitude of any potential bias		collected to answer the specific research question(s). Include discussion of misclassification bias, unmeasured confounding, missing data, and changing eligibility over time, as they pertain to the study being reported.	
Interpretation	20	Give a cautious overall interpretation of results considering objectives, limitations, multiplicity of analyses, results from similar studies, and other relevant evidence	6-7		
Generalisability	21	Discuss the generalisability (external validity) of the study results	6-7		
**Other Information**					
Funding	22	Give the source of funding and the role of the funders for the present study and, if applicable, for the original study on which the present article is based	17		
Accessibility of protocol, raw data, and programming code		..		RECORD 22.1: Authors should provide information on how to access any supplemental information such as the study protocol, raw data, or programming code.	17

Supplementary Table S2. Variable definitions

Baseline characteristics	
Smoking status	Yoshiki 1 has the Brinkman index that is the number of cigarettes smoked per day multiplied by the number of years of smoking. We dichotomized it based on 0 or >0.
Home oxygen therapy	At least one following receipt within 6 months before the index date (procedure code of at-department procedure master for the medical service fee):<br><br>Procedure code<br>114003710, 114004310, 114004910, 114005010, 114005410, 114005510, 114006110, 114006210, 114006310, 114006810, 114009610, 114011110, 114011210, 114040710, 114040810, 114041210, 114041310, 114041610, 114041710, 114042770, 114043670, 114045470, 114045670, 114053150, 114055550, 114055650, 114055850, 114055950, 114056350, 114061510, 114062510, 114062610
Systemic steroid	Oral or parenteral corticosteroid administration with methylprednisolone equivalent dose of ≥5 mg for ≥2 weeks within a month preceding admission.<br><br>Medication code<br>2456001F1019, 2456001F2015, 2456400D1016, 2456001F1019, 2456001F1019, 2456001F1019, 2454002S1122, 2456001F2015, 2456001F1019, 2454004F2090, 2454002F3020, 2456001F2015, 2456001F1019, 2456003F2030, 2454404A2010, 2456003F2022, 2452400D1106, 2454002F1183, 2454004Q1078, 2456001F3011, 2452002F1030, 2454004F2065, 2454404A4020, 2452400D1050, 2456003F1034, 2454002F2023, 2454004F2081, 2456002B1062, 2456400D1032, 2454405A5021, 2454405H6026, 2454004F2103, 2456001F1019, 2456400D1016, 2456405D2015, 2456001F2015, 2454401A3020, 2456001F3011, 2454002F1035, 2456400D2012, 2454002S1157, 2456001F1019, 2454405A2022, 2454004B1040, 2456400D2039, 2454004Q1051, 2452400D3036, 2454404A1013, 2456400D3019, 2454004F2ZZZ, 2456405D1019, 2452400D4040,

	2454004B1032, 2456001F1019, 2456001F1019, 2456400D4023, 2454404A3016, 2454002S1149, 2456001F2015, 2456001F1019, 2454405A4041, 2456400D2012, 2454002F1175, 2456001F3011, 2456405D2015, 2452002F1022, 2454405H2020, 2452001F1036, 2456400D3035, 2452402A1087, 2454004B1059, 2454004B1067, 2456400D4082, 2456001F2015, 2454400C4023, 2452402A5015, 2456001F1019, 2456405D3011, 2456400D3019, 2456001F1019, 2456002B1054, 2456001F3011, 2452400D6043, 2454004F2014, 2454003F1030, 2454002S1041, 2456001F1019, 2452402A5104, 2454407A1025, 2454002F1159, 2452402A5074, 2454001F1022, 2454404A4039, 2452001F1028, 2456400D2080, 2456400D3086, 2456402C3020, 2456001F1019, 2454002F1108, 2454402A2029, 2454404A1021, 2454002F1167, 2452400D6086, 2454002S1ZZZ, 2456402C2024, 2456402C1028, 2456003F1026, 2456001F1019, 2456001T1012, 2456001F1019, 2452400D1114, 2452402A5112, 2452400D7040, 2452400D6094, 2456001F1019, 2456400D2098, 2456400D2101, 2452402A2040, 2452400D1084, 2454405H3027, 2456400D1059, 2454404A3067, 2456400D2012, 2456400D2020, 2456400D1024, 2456400D3027, 2456405D1027, 2452400D1092, 2454405H1032, 2456405D2023, 2454404A1064, 2456405D2015, 2454405H5038, 2452400D1076, 2454405A3029, 2454404A1080, 2456400D1067, 2454405H1024, 2456400D2071, 2454405H3035, 2456400D3116, 2454405H4023, 2452400D1041, 2452400D3052, 2452400D6060, 2454405A1034, 2454405A4033, 2454404A2060, 2456400D1083, 2454405A1042, 2454405A3037, 2454404A2087, 2452400D6078, 2454402A3033, 2456400D3094, 2452400D4032, 2456400D3078, 2454408C1020, 2456405D1019, 2454405H5020, 2456400D4074, 2454404A2036, 2456402C2040, 2456402C1044, 2456400D4066, 2452400D4024, 2454402A2037, 2454402A3025, 2456405D3011, 2456400D2110, 2454407A1033, 2454400C1024, 2452402A1095, 2454404A1030, 2456400D3019, 2456400D1040, 2454405A6028, 2456400D3108, 2452400D6035, 2456400D4090, 2452402A5090, 2456400D4040, 2454400C2020, 2454404A3083, 2456400D1016, 2454404A1013

Immunosuppressive agents	Any oral or parenteral immunosuppressive agents usage for ≥2 weeks within a month prior to admission.<br><br><u>Medication code</u><br>3999004M4028, 3999016M1021, 3999016F1030, 3999016F1049, 3999017M1026, 3999002F2027, 3999014M1022, 3999014M2037, 3999004M3021, 3999004M5024, 6399423F1026, 3999004M4109, 3999004M5105, 3999004M4010, 3999004M5016, 4291024M1024, 4291038M2022, 3999016F1073, 6399414A1038, 3999016F1022, 3999016M1056, 3999043F1020, 3999014F2054, 3999005F1016, 3999002F1020, 3999014M2029, 3999016M1ZZZ, 3999014N2024, 3999022F1028, 3999022F2024, 3999014N1028, 3999014N3020, 6399418D1032, 3999005F1016, 3999014M3025, 3999002F4020, 3999444G4021, 4291019M1023, 3999448G2027, 3999448G3023, 3999014M1030, 3999014D1022, 3999004M4117, 3999004M3110, 3999004M3145, 3999042F3028, 3999429G2024, 3999053F2020, 3999042F1025, 3999042F2021, 3999004S1036, 3999002F2035, 3999448G1020, 3999016F1014, 4291019M2020, 4291038M4025, 6399421G1022, 3999016M1080, 3999038F1029, 3999445D2027, 3999445D1020, 3999053F1023, 3999048G2024, 3999004M5091, 3999005F1040, 4291038M3029, 3999029M1029, 3999004M4087, 3999004M5083, 1190402A1028, 1190024M1028, 3999043F2026, 3999004S2032, 3999017M1042, 2399403F1039, 3999034F1020, 3999426G4023, 3999426G6026, 2399405F1020, 3999025F1021, 3999014D2029, 3999004M5113, 3999439G1021, 3999442G2020, 3999016F1ZZZ, 3999004M3102, 6399424A1023, 6399427A1027, 6399427A3020, 3999004M1029, 3999004M2025, 3999020F1029, 3999020F2025, 6399421F1027, 3999004M4095, 3999005F1016, 2399403F1047, 3999017M1ZZZ, 3999014F4049, 3999017M1050, 3999016M1064, 3999014M1ZZZ, 3999014M2ZZZ, 4291038M1026, 3999004M3064, 3999004M5075, 4291024M2020, 6399428G1024, 3999406A1024, 3999014M2061, 3999004M5121, 3999446G1021, 3999002F2ZZZ, 3999029M1037, 3999014F3042, 3999016M1072, 3999048G1028, 3999014M2070, 3999014M1073, 3999014M1065, 3999014F2020, 3999005F1016, 3999444G2029, 3999004S2024, 3999437G1022, 3999441G1029, 4291451A1028, 4291451A2024,

	3999439G2028, 3999014F4022, 3999057F1021, 3999437G2029, 3999434A1026, 3999002F1055, 3999046F2020, 3999017M1034, 3999004M4079, 6399414A1020, 3999451G4021, 3999445G2023, 3999462A1028, 3999002F2051, 3999014M1049, 3999056M1026, 6399418D1024, 3999004M3099, 3999016F1065, 3999004M4141, 3999450G2024, 3999459G3021, 3999014F2038, 3999004M5ZZZ, 3999046F1023, 3999004M4ZZZ, 3999014F1058, 3999014F4ZZZ, 3999014M1057, 3999014F1023, 3999467G1023, 3999468G1028, 6399430A1029, 1190024M2024, 6399413A1025, 3999048G4027, 2399407A1021, 3999450G3020, 3999025F1030, 3999014F3026, 3999016M1013, 3999417D1037, 3999002F1039, 2399404F1033, 4291035F1023, 2399018F1024, 3999002F3023, 4900400X1021, 3999014F1031, 3999417D1029, 3999426G2020, 3999457G3022, 3999448G4020, 3999463G4024, 3999426G7022, 3999016F1057, 3999016M1099, 3999002F1ZZZ, 3999456G1025, 3999014M3033, 3999451G4030, 3999451G2037, 3999439G4020, 2399404F1025, 3999450A1025, 4291019M3026, 3999004M3ZZZ, 6399403D2024, 3999014M2053, 3999051F1024, 3999051F2020, 3999004M3013, 3999017B1025, 3999004M5148, 3999022F3020, 3999004M4125, 3999014F4030, 3999004M3013, 3999434D1022, 6399427A2023, 3999004M3080, 3999014M2045, 3999442G1023, 3999048G3020, 3999450G4027, 3999014F3ZZZ, 3999014F6025, 3999016M1048, 3999463G3028, 3999016M1102, 3999444G3025, 3999014F6033, 6399409A1029, 3999016F1014, 3999014F2046, 3999004M5016, 3999004S1028, 3999424G6027, 3999017M1069, 3999020F3021, 3999451G3033, 3999445G1027, 3999444G1022, 3999004M5059, 3999014F1040, 4291428A1029, 3999424D2025, 3999468G2024, 3999468G3020, 3999468G4027, 2399406F1024, 3999451G2029, 3999002F2043, 3999004M4010, 3999014M3076, 3999025F1ZZZ, 3999439G3024, 6399429A1026, 6399431A1023, 3999004M5040, 3999004M3129, 3999004M3048, 3999004M4052, 3999004M5067, 3999424G1025, 3999424D1029, 3999424G2021, 3999424G3028, 4291407A1027, 4291407A2023, 4291407A1035, 3999406A1032, 2399402F1026, 4291439A1027, 4291439A2023, 4291407A2031, 6399421A2026, 6399421A3022, 3999429D1021, 3999433G1024, 6399421G2029, 3999426G1024, 3999431A1022, 3999433G2020,

	3999429G1028, 3999426G3027, 3999426G5020, 3999416A2024, 3999431G1025, 3999416A1028, 6399421A1020, 3999450G1028, 3999424G4024
Dyspnea score (Hugh–Johns classification)	Each item was defined as follows:<br>I: “Is the patient's breath as good as that of other men of his own age and build at work, on walking, and on climbing hills or stairs?”<br>II: “Is the patient able to walk with normal men of own age and build on the level but unable to keep up on hills or stairs?”<br>III: “Is the patient unable to keep up with normal men on the level, but able to walk about a mile or more at his own speed?”<br>IV: “Is the patient unable to walk more than about 100 yards on the level without a rest?”<br>V: “Is the patient breathless on talking or undressing, or unable to leave his house because of breathlessness?”
Oxygen use on admission	Oxygen therapy on admission or the next day.<br><br>Procedure code: 140005610
Mental status	In our statistical analysis, we defined the cut-off value as follows:<br>High: 0 < score<br>Low: score = 0<br><br>0: Normal<br>1-digit code: the patient is awake without any stimuli, and is:<br>1: Almost fully conscious<br>2: Unable to recognize time, place, and person<br>3: Unable to recall name or date of birth<br>2-digit code: The patient can be aroused (then reverts to previous state after cessation of stimulation):<br>10: By easily being spoken to (or is responsive with purposeful movements, phrases, or words)<br>20: With a loud voice or shaking of shoulders (or is almost always responsive to very simple words like yes or no or to movements)<br>30: Only by repeated mechanical stimuli<br>3-digit code: The patient cannot be aroused with any forceful mechanical stimuli, and:

	100: Responds with movements to avoid the stimulus<br>200: Responds with slight movements, including decerebrate and decorticate posture<br>300: Does not respond at all except for changes in respiratory rhythm
Activities of daily living (Barthel index)	The final score is total score * 5 to get a point score out of a 100. In our descriptive analysis, we defined the cut-off value as follows:<br>Bowels<br>0: Incontinent (or needs to be given enema)<br>1: Occasional accident (once/week)<br>2: Continent<br>Bladder<br>0: Incontinent, or catheterised and unable to manage<br>1: Occasional accident (max. once per 24 hours)<br>2: Continent (for over 7 days)<br>Grooming<br>0: Needs help with personal care<br>1: Independent face/hair/teeth/shaving (implements provided)<br>Toilet use<br>0: Dependent<br>1: Needs some help, but can do something alone<br>2: Independent (on and off, dressing, wiping)<br>Feeding<br>0: Unable<br>1: Needs help cutting, spreading butter, etc.<br>2: Independent (food provided within reach)<br>Transfer<br>0: Unable – no sitting balance<br>1: Major help (one or two people, physical), can sit<br>2: Minor help (verbal or physical)<br>3: Independent<br>Mobility<br>0: Immobile<br>1: Wheelchair independent, including corners, etc.<br>2: Walks with help of one person (verbal or physical)<br>3: Independent (but may use any aid)<br>Dressing

	0: Dependent<br>1: Needs help, but can do about half unaided<br>2: Independent (including buttons, zips, laces etc.)<br>Stairs<br>0: Unable<br>1: Needs help (verbal, physical, carrying aid)<br>2: independent up and down<br>Bathing<br>0: Dependent<br>1: Independent (or in shower)
Exposures	
Empirical anti-pseudomonal antibiotics	Intravenous anti-pseudomonal antibiotics administered on admission or the next day as the intervention, regardless of the dose.<br><br>Medication code<br>2634710M1085, 6241010F2027, 6241013F3027, 6241013F3051, 2647709M1102, 6135001F2025, 6241013F1024, 6241018F1027, 1319742Q2027, 6241013F2055, 2647709Q1040, 1319722M1013, 1319742Q1250, 1319722M1013, 6123404A1060, 6241017F1022, 2634710M1034, 6131403D2019, 6241013F3019, 1319749Q1030, 1319742Q1039, 1319742Q2019, 6135001R2110, 2634710M1077, 6241010C1024, 2647709M1137, 2634710M1050, 2647709N1060, 1319742Q2019, 6241013F2020, 1329706Q1039, 2634710N1030, 1329706Q1020, 1319722M1056, 1317708Q1037, 6132418F2110, 2647709M1145, 1319800M1023, 1319742Q1ZZZ, 6241013F2012, 1319801Q1020, 1319742Q1020, 6241008F2020, 6132418F2021, 1319722M1021, 1319727Q1174, 6241013F3019, 6123402A3012, 1319722Q1112, 1319722Q1023, 2647709M1064, 1319727Q1026, 1319722Q1163, 2634710M1093, 2647709M1110, 6241013F2306, 1319742Q1144, 2647709N1078, 6241013C2032, 6139400D1068, 6241013F3ZZZ, 1319742Q2019, 1319751Q1020, 6241006F1121, 6241014F1029, 6241013F1229, 6241013C2024, 1319749Q1022, 1319742Q1152, 2634710M1ZZZ, 6131403D1195, 6241013F1199, 6131403D2191, 6131403D1012, 6241013F2250, 6241013F3256, 1319742Q2132, 6241013F5020, 6241013F2209, 6241013F3205, 2634710M1069, 6241013F1210, 6241015F1023, 1319742Q2019,

	6135001R1025, 6135400F2058, 1319742Q1233, 6241008F1112, 6132418F2161, 1319742Q2108, 1329706Q1012, 6119400A3037, 6241013C1028, 6241013F3043, 6241005F2018, 1319722Q1210, 2647709M1013, 1319742Q2019, 2634710M1026, 6241005F1011, 6132424F2051, 6241008F2143, 1319742Q2140, 1317708Q1029, 1325703Q1036, 1319802Q2020, 1319751Q1038, 6241008F1023, 6241008F2089, 6241005F1020, 6139400D3028, 6123402A1010, 1319742Q2248, 2647709M1ZZZ, 1319722Q1015, 6123402A3063, 6241013F3019, 2647709Q1015, 6135001F1029, 6241006F1016, 6241013F4023, 1319742Q1217, 6132424F2086, 6135400F3011, 6132418F2030, 6131403D1039, 1319722Q1058, 2634710M1018, 1319742Q2019, 1319722Q1015, 6241008F2011, 1319742Q2019, 1319727Q1158, 6241005F1011, 6241013F2012, 6123402A3012, 6241010C1ZZZ, 2647709Q1031, 6241013F3302, 1319722Q1015, 2634710M1042, 1319742Q1209, 6241008F2ZZZ, 6119400A1085, 6241013F3159, 1319722Q1104, 6241013F3175, 6241008F2011, 6241013F1245, 1319722Q1015, 6134407A3027, 1319722Q2011, 6241005F2026, 6241010C1059, 1319802Q2047, 6241013F1237, 1319722Q1139, 6134407A3019, 6241010F2116, 6241008F2011, 6119400A2090, 6241013F2ZZZ, 6134407A1016, 6241008F2160, 6135400F2201, 6241005F3022, 6241013F3019, 6131403P2015, 1329706Q1012, 6241018F1035, 6131403D1012, 1319802Q2039, 1319742Q2ZZZ, 1319742Q1098, 6241400A1022, 1319734Q1034, 1319742Q1284, 6132418F2048, 6241013F1091, 6119400A2065, 6241013F3019, 2647709N1043, 6123402A3160, 6135400F1116, 6241013F7022, 6241013F3019, 6135001M2130, 6132424F1080, 6119400A2014, 6119400A3029, 6132418F1025, 6241010F2035, 6123402A3225, 6241400A6032, 6132410D2024, 6241013F2152, 2634710N1021, 1319742Q1241, 6241009M1027, 1319722Q1015, 1319742Q2051, 6123402A3080, 6123402A1222, 6241013F3019, 1319815M1020, 1317706Q1097, 6134407A2101, 1319722M1ZZZ, 2634710M1018, 6241013F3264, 2647709N1019, 6135001M2016, 6241010C1032, 1329709Q1032, 6241010F2086, 6241013F2292, 1319742Q1136, 6123404A1116, 6241009M1035, 6241008F1015, 1319742Q1080, 6139400G2036, 6123402D1040, 2647709M1048, 6241018C1020, 6241013S2029, 6132425D2012, 6241008F1ZZZ,

	1319727Q1115, 6241400A5010, 6241013F3108, 6241013F3230,<br>6131403D2035, 6132425D1040, 1329706Q1047, 1319722Q1015,<br>6241013F1270, 6241013F6026, 6123404A2023, 6241013F3086,<br>6241008F2178, 1319801Q1062, 1319722Q1ZZZ, 1319742Q1063,<br>6119400A4017, 1319722Q1015, 6123402A3012, 6241018F1ZZZ,<br>6241400A6040, 1325703Q1028, 6135400G2045, 6139505G2037,<br>6139505F4069, 6132418F1130, 6119400A4017, 1319742Q2094,<br>6241009F1028, 6131403D2140, 6241005D1029, 6135400F1027,<br>1319734Q1042, 6241013F3019, 6241013F2012, 6241010F1020,<br>6132418F1068, 1319800M1015, 6241005F2310, 6241013F2012,<br>1319722M1030, 6241008F1015, 6135001R2013, 6139500F2012,<br>6241013F1083, 6132418F2013, 6241013F2012, 2647709N1ZZZ,<br>6241013F2012, 1317706Q1100, 6241010F2043, 6131403P1019,<br>6241013F1261, 1317706Q1020, 6134407A2012, 6241010F2078,<br>6241013F2080, 1319742Q1047, 6241013F2012, 6119400A4017,<br>6241013F2233, 6119400A2014, 6134407A2012, 6241008F1040,<br>6241013F1075, 1319742Q1276, 6241013F2179, 6241013C2ZZZ,<br>6241013C1052, 6139500F2071, 6131403D2248, 1319722Q1015,<br>6241008F1015, 6241013F3329, 6241010F1055, 6241400A3076,<br>2639803X1027, 6241013F1121, 1319727Q1018, 6125400D4029,<br>2647709Q1023, 6132424F1020, 6129400F1020, 6241013F1180,<br>1319722Q1015, 6241013F2012, 6241013S1022, 6241402G1105,<br>1319742Q1195, 6132418F2188, 6241010F2ZZZ, 6241013F3299,<br>6139400G1056, 6134407A2098, 1319722Q1015, 6139400D1106,<br>1319742Q1306, 1319734Q2030, 6241006F1016, 1319727Q1018,<br>1319727Q1190, 1329706Q1ZZZ, 6123402A1010, 6241005F2018,<br>6241006F1016, 1319734Q1026, 6241006F1016, 1317706Q1119,<br>6169700G1025, 6135001M2024, 1319742Q2124, 6135400F2147,<br>6135400F2015, 6135400F2031, 1319722Q1015, 6241010F2124,<br>6241013F2047, 1319722Q1015, 6241013F1288, 6135400F3097,<br>6241013F1032, 6123402A1087, 6241013F2012, 6241013F3140,<br>6119400A2057, 6241010C1040, 6241010C1067, 6123402A1095,<br>6241013F2012, 6135400F1019, 6119400A1050, 6241013F1130,<br>2647709Q1ZZZ, 6241013C1095, 1319742Q2019, 6241400A4099,<br>6241010F2051, 6241013F3094, 1319722Q1015, 1319742Q2019,<br>6241013F1202, 1319742Q1179, 6123402A1010, 1329709Q1024,

	6241013F1156, 6241013F3035, 1319722Q1015, 1319722Q1015, 6241010F1098, 6241013F2039, 6241013F3221, 6241010F2019, 6119400A2081, 1319727Q1220, 6241013F1148, 6241013F2322, 6241006F1016, 6131403D1136, 6241008F1139, 6241005F1ZZZ, 1319742Q1055, 6241010F1110, 1319742Q1187, 1319722Q1066, 6241013F3019, 6135400F2155, 6135400F3151, 1319722Q1015, 6241013F2101, 6139505F3054, 6241400A4013, 6241006F1016, 6241005F2301, 1319727Q1140, 6241005F2018, 6241008F1015, 2647709N1051, 6241013F3019, 1319727Q1ZZZ, 6135001M1052, 1319722Q1015, 1319742Q2019, 6119400A3045, 6241013F2012, 6241010F2060, 6241008F2011, 2647709M1013, 6241010F2094, 6241008F1147, 1319722Q2011, 6135001F2ZZZ, 6241005F2018, 1319742Q2116, 6139400G2028, 6241013F1253, 1319727Q1018, 6241008F1015, 6241010F1047, 6241013C1079, 6241008F2011, 6241013F1105, 6241008F2038, 6119400A2030, 6135400F1043, 6241013F3191, 6241013C1060, 1319742Q1268, 6241013F2195, 6123404A1094, 6135400F3119, 1319727Q1069, 6241010F2108, 6123700G1026, 6241013F3060, 1319722Q1180, 1319742Q2060, 6241008F2011, 6241010F1080, 1319742Q1225, 6119400A3010, 6135001M1044, 6241005F1275, 6241010F3023, 6135001F1ZZZ, 6241010F2019, 6241013F1059, 1319742Q1128, 6241008F2011, 6132426G4024, 6241010F1039, 6241013F7014, 6241008F1074, 1317706Q1127, 6241013C1087, 6241013F6018, 6241008F2135, 1317706Q1ZZZ, 6241013F1113, 6241013F3019, 6241013F2144, 1319742Q1160, 1319722Q1120, 6131403D2264, 6119400A1018, 6241005F2018, 6135001R2021, 6241006F1016, 6241006F1075, 6241010F1101, 6241005F1267, 1319742Q1101, 6241013F3183, 6241006F1016, 6119400A1093, 6241013F2098, 1319742Q2035, 6241013F2128, 1319742Q1110, 1319742Q2230, 2647709M1072, 6241013F2187, 6241008F1058, 6132424F1071, 6241005F2018, 6241013F1296, 1319722Q1031, 6241013F2063, 6241013F2012, 1319727Q1018, 6241013F1172, 1317706Q1089, 6241008F2070, 1319722M1013, 1317706Q1011, 6131403P1019, 6139400D2030, 6132425D2039, 6139401D1020, 6139500F2160, 6139400D2110, 6139505G1057, 6139505F3127, 6119400A2073, 6139402D1032, 6139505F4115, 6132418F2129, 6132426F2026, 6139503D2023,

	6139400D2021, 6139505F4026, 6131403D2159, 6139400D2129, 6139500G1070, 6139505F4123, 6139500G1054, 6139500G1020, 6135400F2163, 6135400F2198, 6139400D2064, 6139505F4050, 6132425D2020, 6139505G1049, 6139500F2128, 6139400G1064, 6139500F2179, 6139500G1089, 6132426G3028, 6131403P2023, 6139505F4107, 6123402A3110, 6132418F2064, 6123402D1059, 6241402G1024, 6139402D2020, 6241400A5028, 6132424F2027, 6131403D1047, 6123402A1125, 6123402A3128, 6241400A5052, 6123402A3012, 6131403D2230, 6139500F2063, 6139500F2136, 6241400A3025, 6132418F2153, 6131403D2221, 6135400G4021, 6123402D2039, 6139500F2110, 6139400G1030, 6132425D2047, 6131403D1152, 6241400A3041, 6139401G1026, 6123401A2038, 6139500F2098, 6135400F3178, 6241401G2026, 6119400A2014, 6139505F3020, 6241402G1032, 6132418F2056, 6123404A2031, 6134407A2047, 6131403D2043, 6134407A1024, 6135400F3054, 6139500F2020, 6241402A1030, 6139400G1048, 6139400D2056, 6139400D2102, 6139505G1022, 6241400A2029, 6119400A2022, 6134407A3043, 6241401G2042, 6123402A3187, 6135400F1051, 6132424F2078, 6134407A2071, 6139505G1030, 6123404A1086, 6132425G1020, 6139400G1021, 6123404A1108, 6134407A3078, 6132418F2145, 6241400A6024, 6131403D2213, 6139400D1114, 6122400D1028, 6139505F2023, 6139500F2055, 6139400D1033, 6139400D3036, 6139505F3100, 6122400D2024, 6131403D1144, 6123404A1027, 6139503D1027, 6132424F2043, 6135400G1022, 6131403D1217, 6139500F1024, 6132425D1032, 6123402D1067, 6134407A2080, 6139400D1025, 6132410D2032, 6123402A3012, 6123404A2082, 6132418F2137, 6131403D2167, 6123404A1078, 6139500F2144, 6134407A3094, 6131403G1027, 6135400F3208, 6241400A3050, 6123402A1010, 6131403D1225, 6131403D1209, 6135400F1078, 6123402A3047, 6131403P2015, 6139400D2080, 6139505F4093, 6131403P1027, 6123401A3026, 6241401G2034, 6123402A1010, 6123402A1184, 6241401G1046, 6131403D2205, 6241402A1021, 6123402A1044, 6131403D1160, 6139505F1027, 6132424F1047, 6132426F1020, 6139500G1046, 6241402G1091, 6131403D1233, 6241400A3092, 6135400F2228, 6135400F3089, 6131403D1179, 6139402D1024, 6139500F1040, 6123402A1010,

	6139505F3119, 6241400A5036, 6139400D2048, 6123402D2055, 6241402G1083, 6135400F3160, 6241401G3022, 6132418F1041, 6119400A4033, 6123402A1117, 6132418F2099, 6241400A3068, 6119400A1069, 6119400A4025, 6132418F2170, 6139402G1039, 6241400A4021, 6123402D2063, 6123404A1035, 6119400A1026, 6132418F1084, 6132425D1024, 6139400D1122, 6123402A3209, 6139505G2045, 6131403D1241, 6123404A2074, 6123404A2058, 6139400D2099, 6134407A1040, 6139402G1020, 6241401G1020, 6135400G3025, 6123401A1031, 6135400F3224, 6119400A4041, 6135400F3232, 6134407A3019, 6123402D1032, 6139500F1121, 6132418F1092, 6135400F2210, 6123404A2090, 6135400F3216, 6241401G1038, 6131403D2019, 6139505F4085

Supplementary Table S3. Patient characteristics stratified by the presence of bacterial culture results

	Absence of bacterial culture results (N* = 684)	Presence of bacterial culture results (N = 171)
Age (mean (SD[b]))	74.9 (12.4)	76.6 (11.5)
Male	543 (79.4)	137 (80.1)
Number of beds (%)		
≥100–<300	87 (12.7)	0.0 (0.0)
≥300–<500	341 (49.9)	19 (11.1)
≥500	256 (37.4)	152 (88.9)
Source of infection (%)		
Community-acquired	531 (77.6)	145 (84.8)
Nursing-care-acquired	60 (8.8)	11 (6.4)
Hospital-acquired	93 (13.6)	15 (8.8)
Body mass index (%)		
<18.5 kg/m^2	162 (23.7)	26 (15.2)
≥18.5–<25 kg/m^2	325 (47.5)	68 (39.8)
≥25 kg/m^2	119 (17.4)	14 (8.2)
Missing	78 (11.4)	63 (36.8)
Activity of daily living (%)		
Full support	184 (26.9)	27 (15.8)
Partially dependent	74 (10.8)	38 (22.2)
Independent	426 (62.3)	106 (62.0)
Altered mental status (%)	149 (21.8)	49 (28.7)
Missing	5 (0.7)	3 (1.8)
Exercise tolerability (%)		
Low	230 (33.6)	73 (42.7)
Missing	5 (0.7)	1 (0.6)
Immunodeficiency (%)	178 (26.0)	33 (19.3)
Home oxygen therapy (%)	9 (1.3)	3 (1.8)

Smoking (%)	387 (56.6)	114 (66.7)
Charlson Comorbidity Score (median [IQR[c]])	4.0 [3.0, 6.0]	4.0 [3.0, 5.0]
Previous antibiotics use within 90 days before admission	119 (17.4)	29 (17.0)
Dialysis at baseline (%)	4 (0.6)	1 (0.6)
Blood urea nitrogen (%)		
<14 mg/dL	269 (39.3)	38 (22.2)
≥14–<22.4 mg/dL	222 (32.5)	75 (43.9)
≤22.4 mg/dL	185 (27.0)	58 (33.9)
Missing	8 (1.2)	0.0 (0.0)
Serum albumin (%)		
≤2.7 g/dL	109 (15.9)	9 (5.3)
Missing	45 (6.6)	2 (1.2)
Oxygen use on admission (%)	421 (61.5)	109 (63.7)

Abbreviations: *: N = number

Supplementary Figure S1. Balance check after the assignment of propensity score weight

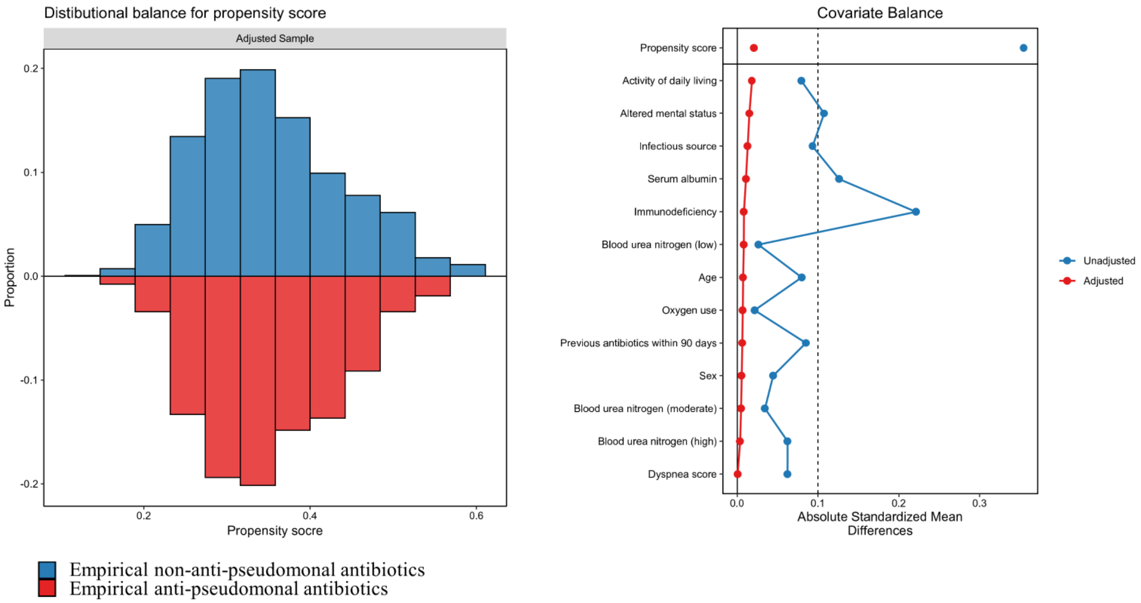

Supplementary Figure S1. This figure visualizes the distributions of the propensity scores and covariates before and after the assignment of propensity score weight. We confirmed the well-balanced distribution.

Supplementary Figure S2. Bias analysis results

$\alpha(p_1)$	$\beta(p_1)$	$\alpha(p_0)$	$\beta(p_0)$	RR_{cd}
36	55	3	30	3
48	48	12	48	4
55	36	24	56	5
36	55	3	30	3
48	48	12	48	4
55	36	24	56	5
36	55	3	30	3
48	48	12	48	4
55	36	24	56	5

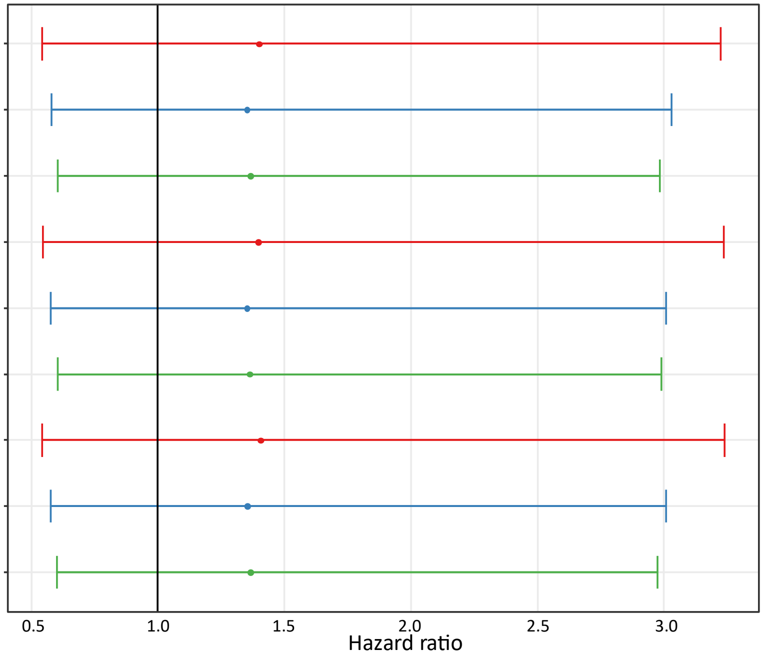

Supplementary Figure S2. This figure summarizes the results of bias analyses. We performed probabilistic bias analyses for a binary unmeasured confounder based on different pairs of parameters: prevalence of among the empirical anti-pseudomonal antibiotics group ($p_1 = \{0.4, 0.5, 0.6\}$) and that among the empirical non-anti-pseudomonal antibiotics group ($p_0 = \{0.1, 0.2, 0.3\}$), and risk ratio of C and death at 90 days, that is $RR_{cd} = \{3.0, 4.0, 5.0\}$. We found the 95% confidence intervals cross the non-significant threshold of 1.
